# Water channel pore size determines exclusion properties but not solute selectivity

**DOI:** 10.1038/s41598-019-56814-z

**Published:** 2019-12-30

**Authors:** Philip Kitchen, Mootaz M. Salman, Simone U. Pickel, Jordan Jennings, Susanna Törnroth-Horsefield, Matthew T. Conner, Roslyn M. Bill, Alex C. Conner

**Affiliations:** 1 School of Life & Health Sciences, Aston University, Aston Triangle, Birmingham B4 7ET UK; 2 Department of Cell Biology, Harvard Medical School, and Program in Cellular and Molecular Medicine, Boston Children’s Hospital, Boston, MA USA; 3 Institute of Physiology, University of Würzburg, Würzburg, Germany; 4 Institute of Clinical Sciences, College of Medical and Dental Sciences, University of Birmingham, Edgbaston, Birmingham B15 2TT UK; 5 Department of Biochemistry and Structural Biology, Lund University, P.O. Box 124, 221 00 Lund, Sweden; 6 School of Biology, Chemistry and Forensic Science, University of Wolverhampton, Wulfruna St, Wolverhampton, UK

**Keywords:** Biochemistry, Membrane biophysics

## Abstract

Aquaporins (AQPs) are a ubiquitous family of transmembrane water channel proteins. A subgroup of AQP water channels also facilitates transmembrane diffusion of small, polar solutes. A constriction within the pore, the aromatic/arginine (ar/R) selectivity filter, is thought to control solute permeability: previous studies on single representative water channel proteins suggest narrow channels conduct water, whilst wider channels permit passage of solutes. To assess this model of selectivity, we used mutagenesis, permeability measurements and *in silico* comparisons of water-specific as well as glycerol-permeable human AQPs. Our studies show that single amino acid substitutions in the selectivity filters of AQP1, AQP4 and AQP3 differentially affect glycerol and urea permeability in an AQP-specific manner. Comparison between *in silico*-calculated channel cross-sectional areas and *in vitro* permeability measurements suggests that selectivity filter cross-sectional area predicts urea but not glycerol permeability. Our data show that substrate discrimination in water channels depends on a complex interplay between the solute, pore size, and polarity, and that using single water channel proteins as representative models has led to an underestimation of this complexity.

## Introduction

Aquaporins (AQPs) form a family of transmembrane proteins that facilitate a rapid cellular response to the osmotic environment. Water or small, neutral, polar solutes are able to move passively through AQPs down an osmotic or solute concentration gradient, thereby greatly increasing the permeability of biological membranes^1^. AQPs are either strictly water-selective (referred to here as wAQPs) or facilitate the additional movement of solutes such as glycerol and urea in addition to water (known variously as aquaglyceroporins, glyceroporins or GLPs). In addition, all AQPs share a remarkable ability to exclude ions and protons from being conducted through the solute pore.

**Figure 1:**
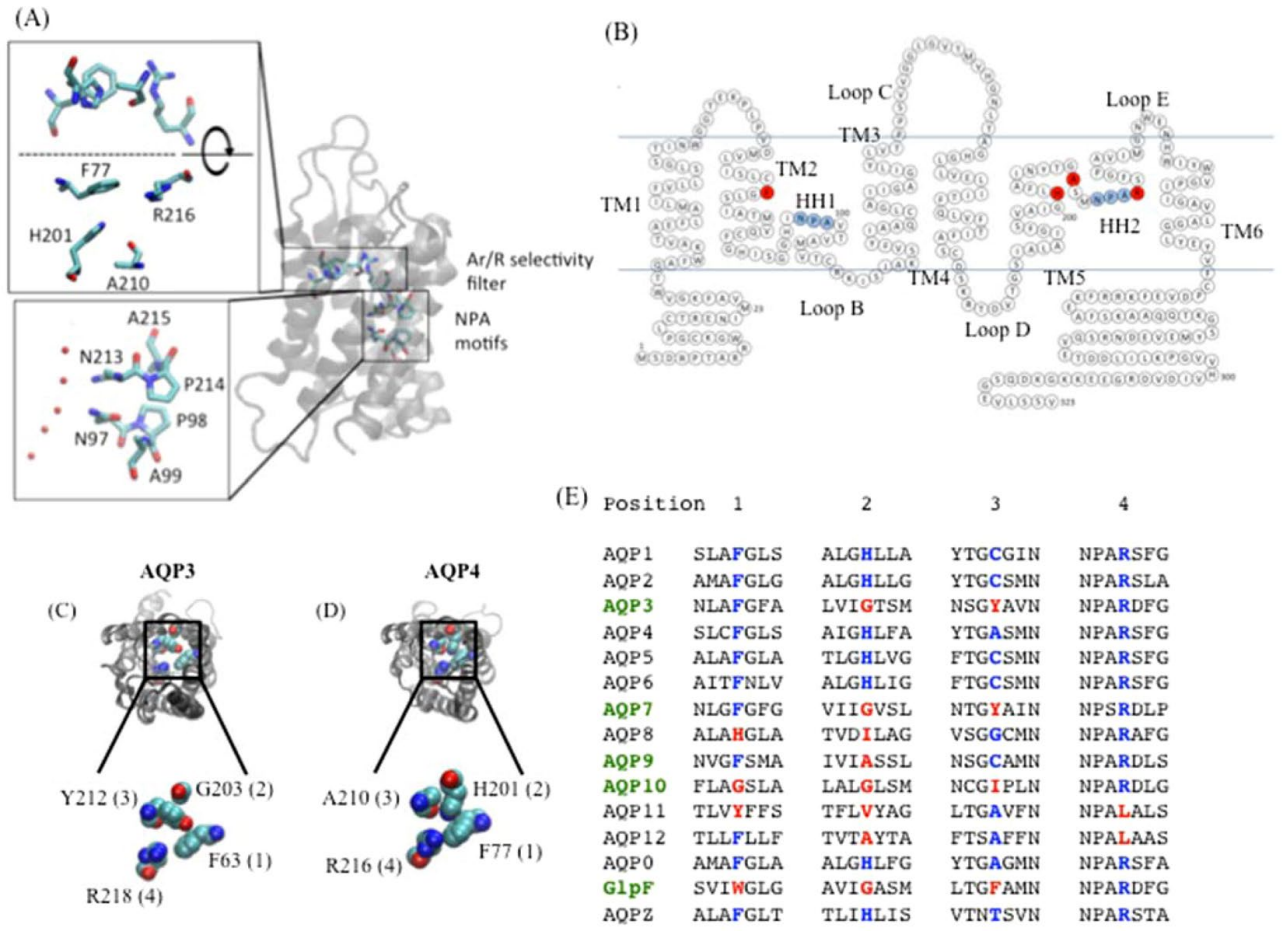
Structure of the AQP ar/R region. (**A**) Location of ar/R filter and NPA motifs in AQP structure, exemplified by AQP4 (PDB entry 3GD8). Red spheres indicate oxygen atoms of co-crystallised water molecules. (**B**) Primary sequence of human AQP4. Residues making up the ar/R selectivity filter are highlighted in red and the conserved NPA motifs in blue. Structures of (**C**) a GLP and (**D**) a wAQP are exemplified by AQP3 (shown is a homology model to the GlpF structure; PDB code 1FX8) and AQP4 (PDB code 3GD8^26^). Numbers in brackets refer to the positions defined in the sequence alignment in (**E**). (**E**) Sequence alignment of human AQPs and two *E. coli* AQPs comparing the four regions contributing to the ar/R region. GLPs are highlighted in green. The conserved residues are highlighted in blue; deviations from this are highlighted in red. Panels B-E are reproduced from P. Kitchen PhD thesis^35^.

The basis for AQP selectivity comes from two conserved structural features within the pore: the NPA region and the aromatic-arginine (ar/R) motif, also known as the selectivity filter (Fig. 1A)^2^. The NPA region, found in the middle of the pore, consists of two copies of an asparagine-proline-alanine (NPA) motif, located at the ends of two short alpha helices formed by loops B and E dipping into the membrane from the intracellular and extracellular sides, respectively (Fig. 1B). The NPA motifs are highly conserved across the AQP family, although variants do exist^3,4^, and are believed to generate the main barrier to proton permeation through AQPs^5^. NPA motifs are not thought to contribute directly to the selectivity differences between wAQPs and GLPs, although subtle structural differences affecting this conserved motif may partially account for quantitative differences in single-channel water permeability between different AQPs.

The second AQP region involved in selectivity, the ar/R-motif, is located towards the extracellular side of the pore and is responsible for determining the difference in solute permeability between wAQPs and GLPs, as well as playing a role in proton exclusion. It is formed by four amino acid residues from disparate locations in the primary sequence (Fig. 1B,C), of which the arginine in position 4 is highly conserved throughout the AQP family. The positive charge presented by this arginine is believed to act as a secondary proton exclusion mechanism^6^ and substitution of the arginine with valine in AQP1 enabled H^+^ permeability^7^. In the less well understood, intracellular “superaquaporins” AQPs 11 and 12, arginine is replaced by leucine^8^. Although functional studies of H^+^ permeability in superaquaporins are yet to be reported, the loss of this arginine residue may suggest roles in intracellular H^+^ homeostasis for AQPs 11 and 12.

The remaining three residues in the ar/R-motif vary between wAQPs and GLPs. In wAQPs, the ar/R- motif is usually comprised of a phenylalanine in position 1, a histidine, in position 2 and a small residue (e.g. cysteine in AQP1 or alanine in AQP4) in position 3. In GLPs, the histidine is typically replaced by a smaller residue (glycine in AQPs 3, 7 and 10, alanine in AQP9 and isoleucine in AQP8), making the presence or absence of a histidine in position 2 the major difference between wAQPs and GLPs. In the crystal structure of the bacterial aquaglyceroporin GlpF, the glycine residue at the equivalent position to the histidine has a structural consequence, allowing the phenylalanine in position 3 to pack in front of it (Fig. 1C). Based on sequence alignment (see Fig. 1D), in the mammalian GLPs this position of the filter region is occupied by a tyrosine (AQPs 3 & 7), cysteine (AQP9) or isoleucine (AQP10).

It is generally believed that the differences in amino acid composition of the ar/R-region determine the specificity between wAQPs and GLPs, primarily by affecting the pore size^2^. This is supported by *in silico* experiments^9^ and an *in vitro* study of rat AQP1 which created urea and glycerol permeable mutants^7^. However, a comparative study of the *E. coli* glycerol channel GlpF and its water-specific counterpart AqpZ failed to introduce glycerol permeability to AqpZ with GlpF-mimicking mutations to the ar/R-region^10^. Moreover, solute hydrophobicity was shown to be anticorrelated with permeability for AQP1 but not GlpF *in silico*^2^.

Structural and functional comparison of GlpF, seven wAQPs and “intermediate selectivity channels” (archeal AQPM and PfAQP from *Plasmodium falciparum*) suggests a correlation between the pore electrostatic potential profiles and glycerol permeability^11^. This strongly suggests that AQP solute specificity cannot be explained by differences in pore size alone.

Our manuscript demonstrates that the generalization of water channel selectivity being based on pore size alone is incomplete. To elucidate the mechanisms behind AQP specificity, we characterized the glycerol and urea permeability of a comprehensive set of ar/R-region mutants of two human wAQPs (AQP4 and AQP1) and the human GLP AQP3. In addition, we also made the first direct and quantitative comparison of the relative glycerol and urea permeability of members of the mammalian GLP family (AQP3, AQP9 and AQP10) in live mammalian cells. Based on these functional data and *in silico* structural analysis, we conclude that water channel solute specificity, in particular for glycerol, depends on a complex interplay between the unique properties of the residues that constitute the ar/R-region, the resulting pore size and the structural context in which these residues are situated.

## Results

### Mutagenesis of the ar/R region of AQP4, but not AQP1, creates channels that are selective for either urea or glycerol

Previous studies of rat AQP1 showed that increasing the diameter of the rat AQP1 pore through substitution of H180 of the ar/R motif to alanine allows the passage of urea. Increasing the diameter further (through the double substitution F56A/H180A) allows passage of both urea and glycerol, with the urea permeability approximately two-fold higher than the glycerol permeability, whilst the water permeability was unchanged^7^.

**Figure 2:**
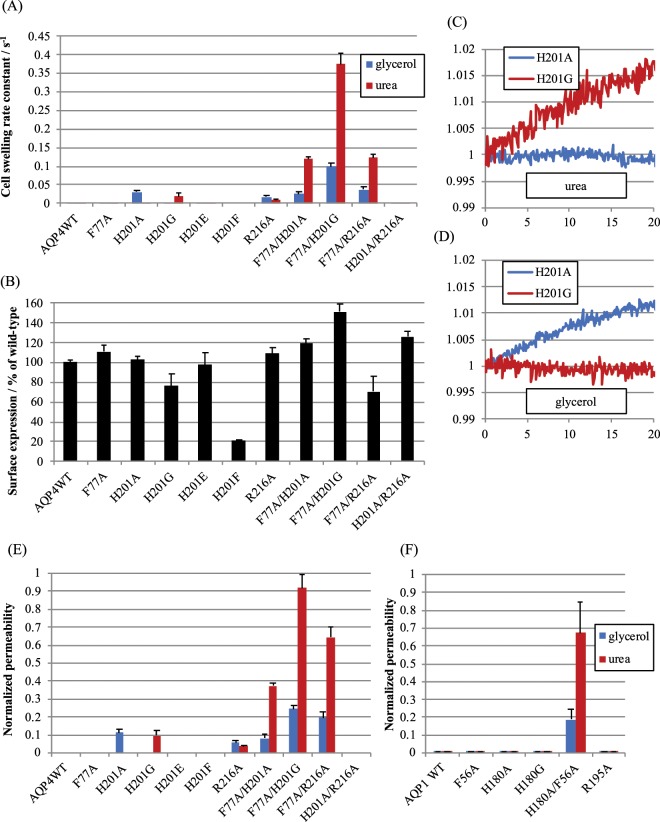
Relative single channel glycerol and urea permeabilities of AQP4 selectivity filter mutants. (**A**) Cell swelling rate constants of HEK293 cells transfected with AQP4 mutants, calculated from calcein fluorescence time-series data. (**B**) Cell-surface expression of AQP4 mutants in HEK293 cells measured by cell-surface biotinylation and neutravidin ELISA. (**C**,**D**) Representative calcein fluorescence timeseries from HEK293 cells transfected with AQP4 H201A or H201G and exposed to iso-osmotic solutions containing 150 mM of urea or glycerol. (**E**) Cell swelling rate constants from (**A**) above normalized to surface expression and then normalized to AQP3 glycerol permeability. This gives a relative single channel permeability in units in which AQP3 glycerol permeability is 1. (**F**) Normalized permeabilities for the analogous mutants of AQP1 for comparison. Panels A, B, D and F are adapted from P. Kitchen PhD thesis^35^.

To investigate whether substitution of the analogous residues in human AQP4 (F77, H201 and R216) has the same effect, we generated six AQP4 selectivity filter single substitution mutants, F77A, H201A, H201G, H201E, H201F, R216A, and four double substitution mutants, F77A/H201A, F77A/H201G, F77A/R216A and H201A/R216A, using site-directed mutagenesis. These mutants were transiently transfected into HEK293 cells, chosen for their high transfection efficiency and low intrinsic glycerol/urea permeability. Cell swelling with iso-osmotic glycerol or urea solutions was measured using an adaptation of the plate-reader-based calcein fluorescence quenching method^12,13^ to quantify glycerol and urea permeability in live mammalian cells (Fig. 2A). Surface expression was measured by cell-surface biotinylation (Fig. 2B). Of the 10 mutants that were studied, only AQP4 H201F had reduced protein expression compared to WT AQP4, which was further confirmed by Western blot (data not shown). This may be due to an interruption of protein folding by the introduction of steric clashes in the pore between several bulky hydrophobic amino acid side-chains.

Mutagenesis of H201 to different amino acid residues selectively created channels with a preference for either urea or glycerol but not both (Fig. 2A,C,D,E). Substitution to alanine (H201A) conferred glycerol but not urea permeability (Fig. 2C,D). This contrasts with the urea permeability reported for the analogous mutation in rat AQP1 (H180A) expressed in *Xenopus* oocytes, although we could not reproduce this with human AQP1 H180A in mammalian cells (Fig. 2F). Conversely, glycine substitution in AQP4 (H201G) conferred urea permeability but not glycerol permeability (Fig. 2C,D). We have previously demonstrated that these substitutions do not alter AQP4 water permeability^13^.

Substitution of the selectivity filter arginine residue with alanine (R216A) conferred both urea and glycerol permeability to AQP4 (Fig. 2C), in contrast to data for the analogous AQP1 R195V mutant reported by others^7^, which was impermeable to both urea and glycerol. Our data for AQP1 R195A also showed that the channel was impermeable to both urea and glycerol (Fig. 2D). Interestingly, the AQP4 double mutant H201A/R216A lost the effects of either single substitution despite robust surface expression. We previously demonstrated that the R216A mutation modestly increases AQP4 water permeability by approximately 50%, by preventing the arginine sidechain from transiently occluding the pore^13^.

In contrast to the arginine and histidine mutations, the single substitution of the phenyalanine in position 1 with alanine (AQP4 F77A and AQP1 F56A) had no effect on urea or glycerol permeability of either AQP4 or AQP1 (Fig. 2C,D). The AQP4 double mutants F77A/H201A and F77A/H201G were permeable to both glycerol and urea, with a preference for urea and a P_u_/P_g_ ratio of 4.6 ± 1.1 and 3.7 ± 0.4 respectively. This is similar to the equivalent AQP1 mutant (F56A/H180A), which in our hands had a P_u_/P_g_ ratio of 3.6 ± 1.4 that agrees qualitatively with the value of 1.7 ± 0.6 reported for the analagous mutation to rat AQP1^7^. The double F77A/R216A mutant was also permeable to both solutes, with a similar P_u_/P_g_ ratio of 3.3 ± 0.6. Surprisingly, the H201A/R216A mutant was permeable to neither urea nor glycerol despite the permeability of the single H201A and R216A mutants, and despite robust surface expression measured by cell-surface biotinylation (see Fig. 2B), suggesting the the protein is correctly folded and inserted into the plasma membrane.

### GLP-mimetic mutants of AQP4 are not solute permeable

**Figure 3:**
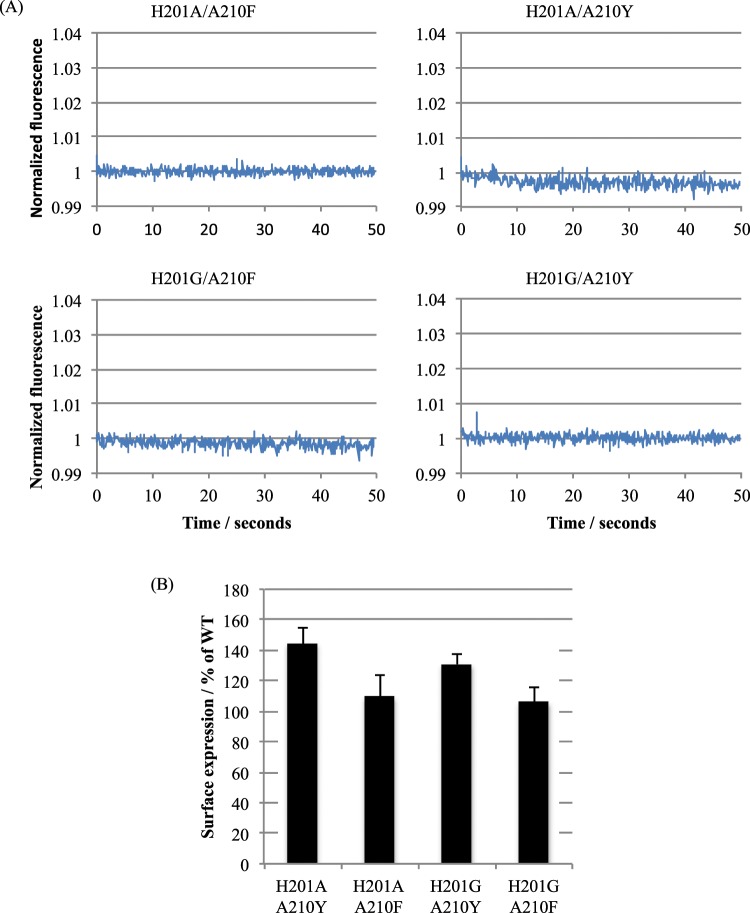
(**A**) Representative calcein fluorescence quenching curves for glycerol permeability of AQP4 GLP-mimetic double mutants. (**B**) Surface expression of AQP4 GLP-mimetic mutants measured by cell surface biotinylation. This figure is reproduced from P. Kitchen PhD thesis^35^.

Having established that the urea and glycerol permeabilities of human AQP4 can be altered by point mutations in the ar/R-motif, we explored whether substituting the ar/R-motif residues found in AQP4 for those found in GLPs would give a channel with GLP-like behaviour. As described above, the main difference between the ar/R-region in wAQPs and GLPs is the histidine in position 2, which is replaced in GLPs by a small residue such as glycine or alanine (Fig. 1). In addition, GLPs typically have an aromatic residue (usually tyrosine or phenylalanine) in position 3, the side-chain of which packs in front of this small residue (as seen in the GlpF crystal structure). The creation of this structural landscape in AQP4 (H201G/A210Y, H201G/A210F, H201A/A201Y, H201A/A210F) did not create a glycerol-permeable channel when expressed in HEK293 cells (representative calcein fluorescence quenching timeseries in Fig. 3A), or a urea-permeable channel (not shown) despite robust surface expression determined by cell surface biotinylation (Fig. 3B).

### AQPs 3, 9 and 10 have different relative permeabilities for glycerol and urea

**Figure 4:**
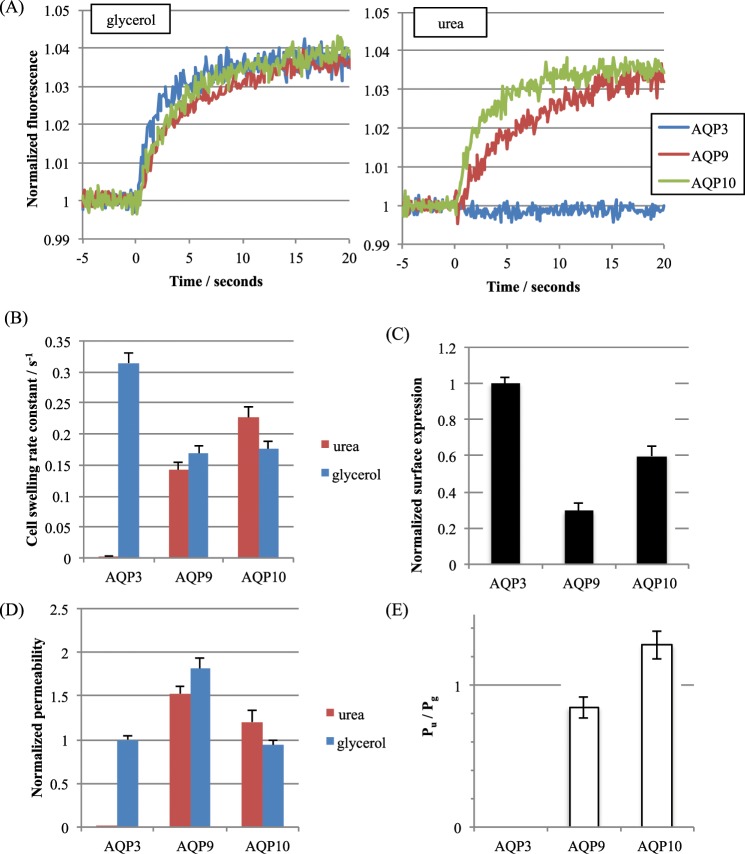
Relative single channel glycerol and urea permeabilities of AQPs 3, 9 and 10. (**A**) Representative calcein fluorescence quenching curves for human AQPs 3, 9 and 10 for glycerol and urea permeability. (**B**) Average cell swelling rate constants from fits to calcein fluorescence timeseries data. (**C**) Surface expression of GFP-tagged AQPs 3, 9 and 10 in HEK293 cells measured by cell surface biotinylation followed by ELISA with an anti-GFP antibody. (**D**) Relative single channel solute permeabilities of AQPs 3, 9 and 10 calculated by normalizing the cell swelling rate constant to surface expression and then to AQP3 glycerol permeability. (**E**) Ratio of urea permeability to glycerol permeability for AQPs 3, 9 and 10. This figure is reproduced from P. Kitchen PhD thesis^35^.

Next, we measured the glycerol and urea permeability of three human GLPs: human AQP3, 9 and 10. All three displayed different, biased selectivity for urea and glycerol (Fig. 4). For AQP3, there is some debate in the literature concerning the urea permeability (see^14^ for a detailed discussion), however in our hands, wild-type human AQP3 expressed in HEK293 cells was not measurably urea-permeable (Fig. 4B). AQP10 had a glycerol permeability of 0.94 ± 0.11 when normalised to AQP3 permeability, whereas the permeability of AQP9 to glycerol was almost two-fold higher at 1.8 ± 0.2 (Fig. 4D). In contrast to AQP3, AQP9 and AQP10 were permeable to both glycerol and urea but were oppositely biased, with ratios of urea permeability to glycerol permeability (P_u_/P_g_) of 0.84 ± 0.01 and 1.29 ± 0.03, respectively (Fig. 4E). These were both significantly different from an unbiased permeability ratio of 1, determined by one-sample t-tests followed by Bonferroni correction for multiple comparisons (p = 0.02 and p = 0.01, respectively).

### The urea and glycerol permeabilities of AQP3 can be altered independently

**Figure 5:**
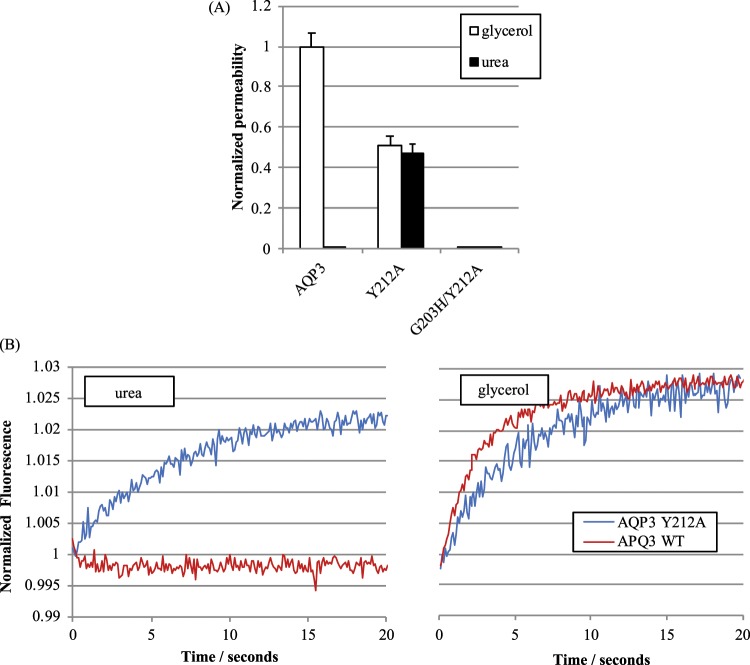
Relative single channel glycerol and urea permeabilities of AQP3 selectivity filter mutants. (**A**) Solute permeabilities of wild-type AQP3 and the selectivity filter mutants Y212A and Y212A/G203H normalized to surface expression and then to AQP3 glycerol permeability. (**B**) Representative calcein fluorescence quenching curves of wild-type AQP3 and the Y212A mutant. This figure is adapted from P. Kitchen PhD thesis^35^.

To investigate whether mutations in the ar/R-motif of AQP3 affect its ability to discriminate between glycerol and urea, we constructed several point mutants and measured glycerol and urea permeability. Substitution of the tyrosine residue in position 3 for alanine (Y212A) conferred measurable urea permeability, whilst concomitantly reducing glycerol permeability to approximately half that of the wild-type (Fig. 5A,B). Combining the Y212A mutation with G203H (G203H/Y212A), to mimic the histidine residue present in wAQPs in position 2, produced a channel impermeable to both urea and glycerol (Fig. 3A). The single G203H mutation caused a reduction in protein expression and surface expression comparable to the AQP4 H201F mutant (data not shown). Again, this was suspected to be due to interrupted protein folding caused by steric clashes in the pore.

### Urea but not glycerol permeability correlates with pore geometry

**Figure 6:**
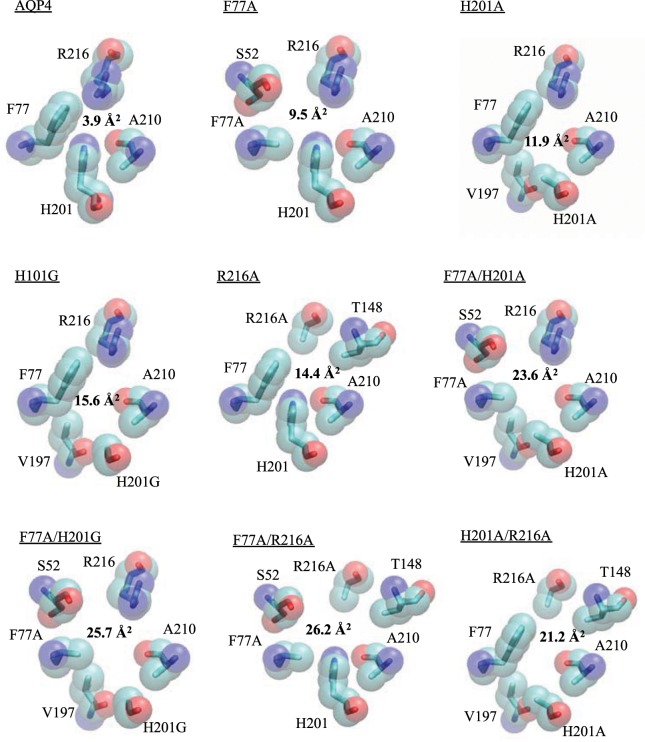
Pore-lining residues of AQP4 mutants at the selectivity filter. Models were constructed by *in silico* mutagenesis of the AQP4 crystal structure and using Swiss PDBviewer. Cross-sectional areas (bold) were calculated using SYBL. This figure is reproduced from P. Kitchen PhD thesis^35^.

**Figure 7:**
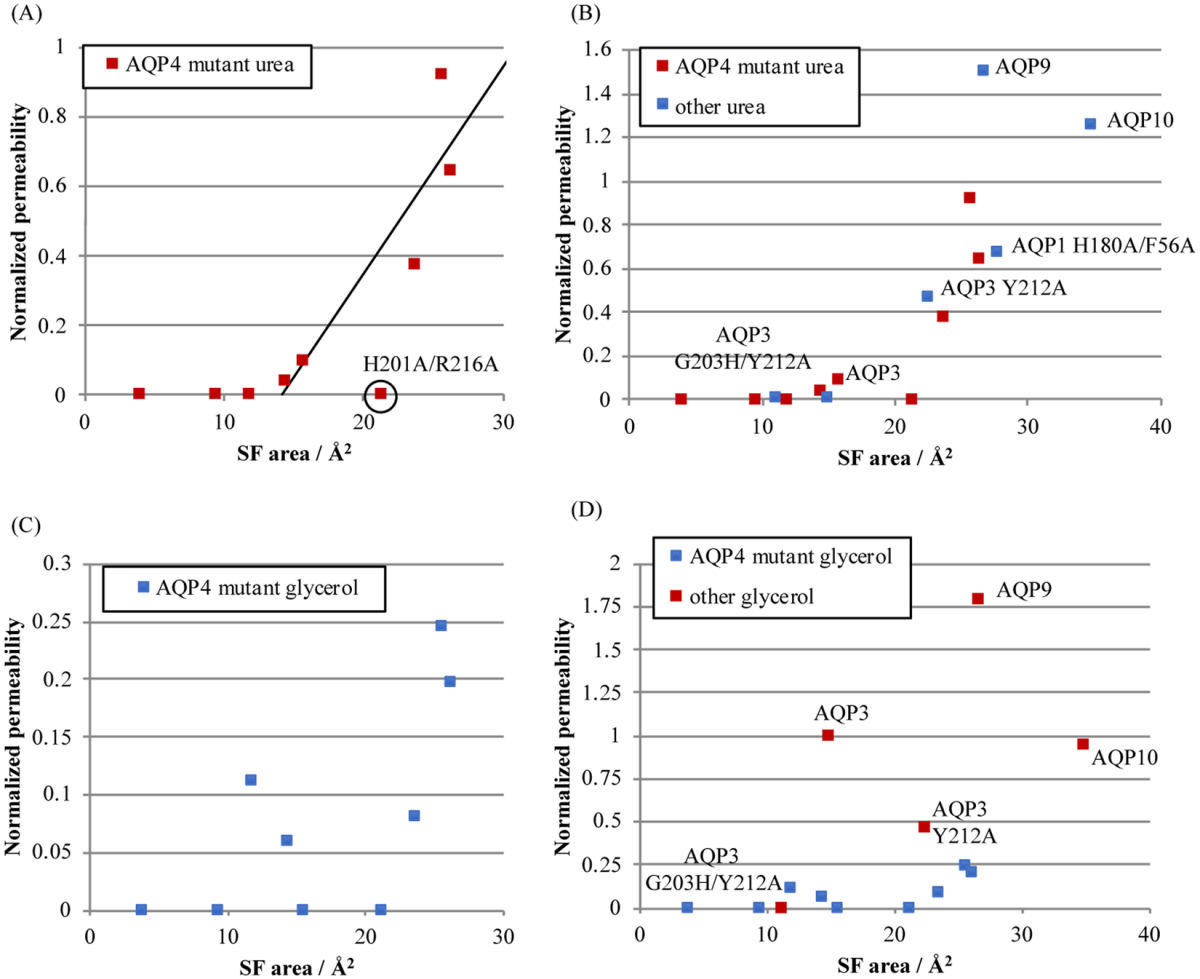
Correlation between selectivity filter (SF) cross-sectional area and (**A**) urea permeability of AQP4 mutants or (**B**) urea permeability of AQP4 mutants and GLPs. The black line in (**A**,**B**) is a linear fit to all AQP4 mutants with non-zero urea permeability; (**C**) glycerol permeability of AQP4 mutants, (**D**) glycerol permeability of AQP4 mutants and GLPs. This figure is adapted from P. Kitchen PhD thesis^35^.

To study the effect of mutations on the structure and pore size of the AQP4 at the ar/R selectivity filter region, we generated models of all of our AQP4 mutants by *in silico* mutagenesis using Swiss PDB Viewer and the crystal structure of human AQP4 (PDB code 3GD8) as a template. The cross-sectional radii and area of the pore at the ar/R-motif was evaluated using the programs HOLE^15^ and SYBYL (Tripos Inc, St. Louis, MO, USA) respectively (Fig. 6 and Table 1). At values greater than ~14 Å^2^, the channel cross-sectional area at the selectivity filter correlated linearly with urea permeability for all AQP4 mutants (R^2^ = 0.83, p = 0.006), with the exception of the urea-impermeable H201A/R216A mutant (circled) (Fig. 7A). Using the AQP4 data as a training set, we then attempted to predict the urea permeability of AQP3, 9 and 10 as well as the AQP3 ar/R-motif mutants. For this purpose, homology models of AQP3, AQP9 and AQP10 were created using Swiss PDB Viewer and the crystal structure of *E. coli* GlpF as the template (PDB code 1FX8). The AQP3 homology model was further used to generate structures of ar/R-motif mutants as described above. As seen in Fig. 7B, the urea permeabilities of 4 of the 5 GLP constructs could be predicted using the AQP4-based linear correlation between urea permeability and cross-sectional area. In contrast, neither the AQP4 mutants nor the GLP constructs showed a clear correlation between cross-sectional area and glycerol permeability. Our data thus suggests that urea permeability, but not glycerol permeability, can be explained by the pore geometry.

**Table 1:** *In silico* channel radii and cross-sectional areas and normalised *in vitro* urea and glycerol permeabilities for AQP4 ar/R mutants.

Construct	Minimum channel radius	Ar/R cross-sectional area	Normalised urea permeability	Normalised glycerol permeability
AQP4	0.90	3.96	0	0
AQP4 F77A	1.54	9.47	0	0
AQP4 H201A	1.57	11.91	0	0.11
AQP4 H201G	1.60	15.64	0.10	0
AQP4 R216A	1.43	14.39	0.04	0.06
AQP4 F77A/H201A	1.64	23.62	0.37	0.08
AQP4 F77A/H201G	1.64	25.69	0.92	0.25
AQP4 F77A/R216A	1.58	26.26	0.65	0.20
AQP4 H201A/R216A	1.60	21.24	0	0

**Figure 8:**
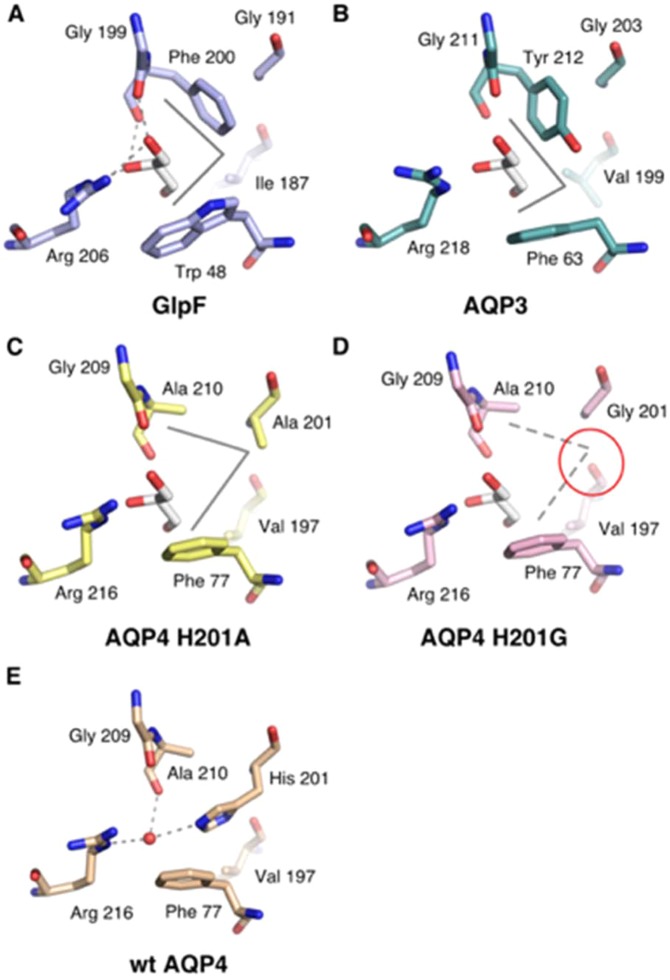
Comparison of ar/R-region in aquaglyceroporins and AQP4 mutants. Structural comparison of the selectivity filter in (**A**) E. coli GlpF (PDB code 1FX8) (**B**) homology model of human AQP3 and (**C**,**D**) *in silico* models of the human AQP4 H201A and H201G. (**E**) The ar/R-region of human AQP4 (PDB code 3GD8) is shown in for comparison. In GlpF (**A**), AQP3 (**B**) and AQP4 H201A (**C**), a hydrophobic corner (grey solid lines) formed by hydrophobic side chains is able to accommodate the glycerol alkyl backbone. In AQP4 H201G (**D**), this corner is broken by the exposure of the backbone carbonyl of Val 197 (red circle). Hydrogen bonds between glycerol hydroxyl groups and residues within the selectivity filter of GlpF (**A**) are depicted as dotted lines. In (**B**–**D**), the position of the glycerol molecule has been superimposed from the GlpF crystal structure.

An unexpected result from our *in vitro* permeability experiments of AQP4 mutants was that the mutation H201A created a glycerol channel, whilst the mutation H201G created a urea channel. We hypothesized that the H201A mutation, together with F77, forms a “hydrophobic corner”, analogous to what is seen in GlpF where the planes of two aromatic residues forms a hydrophobic corner that is suggested to mediate van der Waals contacts with the glycerol alkyl chain (Fig. 8)^16^. In the AQP4 H201G mutant, this corner may be disrupted since the loss of the alanine side-chain could make the V197 backbone carbonyl group solvent accessible and therefore available for hydrogen bonding with water or solute molecules in the pore. This may allow conversion of the H201A glycerol channel to the H201G urea channel, as urea may be able to satisfy the V197 hydrogen bond whereas glycerol cannot. To test this hypothesis *in silico*, we generated 50 ns molecular dynamics trajectories of H201A, H201G and wild-type AQP4 tetramers using Gromacs. In wild-type AQP4 and the H201A mutant, no hydrogen bonds were observed between the V197 backbone and water molecules in the pore, using the Hbonds plugin for VMD with a 3 Å and 20**°** hydrogen bond cut-off. In contrast, in the H201G mutant, we found hydrogen bonds between V197 and water molecules in all four monomers, with an average occupancy of 24.7 ± 5.4% (using the same 3 Å and 20**°** cut-off), with the error estimated as the standard deviation over the four monomers (Fig. S1A). For comparison, we measured the hydrogen bond occupancy of water molecules with the two asparagine residues forming the NPA motifs. These were 55.7 ± 7.0% for N97 and 58.2 ± 10.2% for N213. A representative simulation snapshot in which the V197 side-chain hydrogen bond is occupied is shown in Supplementary Fig. 1B.

## Discussion

Current understanding of water channel structure and function is informed by data from model family members, primarily AQP1 and GlpF. For large protein families with high sequence, structural and functional homologies, the characteristics of model proteins may often be generalised to the whole family. In the case of the highly-homologous family of water channels, the proposed mechanism for solute selectivity is largely derived from comparing structural details of the water-specific channel AQP1 to those of GlpF from *E. coli*, which is also permeable to larger solutes such as glycerol and urea. Based on these studies, it is now widely-accepted that any neutral polar solute will pass through the pore of a water channel as long as the selectivity filter is wide enough.

The work presented here clearly demonstrates that this generalization is incorrect. Instead, the location of specific residues in the selectivity filter of mammalian AQPs and the interplay between them affects solute exclusion and specificity, with the pore size being only one of several contributing factors. Using permeability studies in transfected mammalian cells, we show that in human AQP4, both the histidine (position 2) and arginine (position 4) residues of the ar/R selectivity filter region are crucial for neutral solute exclusion while the phenylalanine (position 1) is not. Specifically, AQP4 H201A and H201G formed glycerol and urea selective channels respectively while AQP4 R216A was permeable to both (Fig. 2C). This is in striking contrast to human AQP1 for which the analogous mutations (R195A and H180A/G) were impermeable to both glycerol and urea (Fig. 2D). Similar results were observed in a previous study in *Xenopus* oocytes in which neither the H180A nor R195V mutations to rat AQP1 generated a glycerol or urea permeable channel^7^. Taken together, these data show that analogous single amino acid substitutions in AQP1 and AQP4 give channels with different exclusion properties. This suggests that the molecular details of how these two water-specific AQPs are able to exclude neutral solutes such as glycerol and urea differ, and does not only depend on the exact composition of the ar/R-motif or the pore size. Consequently, relying on AQP1 as a model of water-selective AQPs has led to an incomplete understanding of how the selectivity filter aids substrate discrimination across the whole family.

Of particular note is the surprising result that mutating AQP4 H201 to alanine or glycine formed neutral solute channels with opposite glycerol or urea permeabilities. In the crystal structure of the *E. coli* glycerol facilitator, GlpF, a co-crystallised glycerol molecule had its carbon backbone packed into a corner created by two hydrophobic residues (W48 and F200, Fig. 8). It was suggested that this hydrophobic corner facilitates the preference of GlpF for glycerol over urea because glycerol can have a hydrophobic “face” that can pack into the corner, whereas urea does not. This necessitates energetically unfavourable breaking of urea-protein or urea-water hydrogen bonds when entering the selectivity filter^16^. Indeed, molecular dynamics (MD) simulations of GlpF suggest that the *gauche-gauche* isomer of glycerol, in which the hydroxyl groups are lined up along one “face” of the molecule leaving an opposing hydrophobic face, is strongly favoured (~80%) in the selectivity filter despite making up <10% of the population in bulk solution^17^. Furthermore, in steered MD, a smaller pulling force was required to make *gauche-gauche* glycerol permeate the GlpF channel compared to other isomers^18^. We propose that the AQP4 H201A mutation, in combination with F77, mimics this hydrophobic effect, whereas the glycine mutant, lacking the hydrophobic methyl group side-chain of alanine, does not (Fig. 8). This is supported by our MD simulations, which suggest that this hydrophobic corner is further disrupted by the exposure of the V197 backbone carbonyl group to the pore in the H201G mutant (Fig. S1A). This would generate an extra hydrogen-bonding site within the pore that may provide an energetic penalty disfavouring the packing of the glycerol backbone (which cannot satisfy the bond) into this region of the selectivity filter.

The structural features of the H201G mutant ar/R-region are similar to the selectivity filter region of the Urea Transporter family of dedicated urea channels (see Supplementary Fig. 2A,B). In these channels, the selectivity filter is elongated and composed of three regions, an inner (S_i_) middle (S_m_) and outer (S_o_) filter region, all of which share the same features with hydrophobic residues on facing sides and backbone carbonyls that provide opposing hydrogen bonds to permeating urea molecules (19, 20. Interestingly, the S_m_-region displays strong structural similarities to the conserved NPA-region in AQPs, with two threonine residues in analogous positions as the asparagines residues. In the H201G mutant ar/R-region, the exposure of the backbone carbonyl of V197 breaks the hydrophobic corner associated with glycerol transport, thus creating a selectivity filter with two opposing hydrophobic sides, mimicking those of the urea channels (Fig. 8D). These structural differences provides a plausible explanation for increased urea selectivity of the H201G mutant.

Alternatively, it is also possible that the observed permeability differences are caused by knock-on structural effects of the mutations, leading to proteins with different channel sizes and/or shapes. If this is the case, the structural change is likely to be subtle, given that both of the AQP4 H201 mutants still form functional membrane channels that are expressed at the cell surface and that we have previously shown have identical single-channel water permeability to wild-type AQP4^13^.

The importance of the hydrophobic corner for glycerol permeability is further supported by our studies of human AQP3. In our homology model of AQP3, a tyrosine residue, Y212, occupied a similar position to the phenylalanine (F200) in GlpF, producing a similar hydrophobic corner as that seen in the GlpF crystal structure (Fig. 8). As this corner was previously suggested to facilitate glycerol selectivity in GlpF^16^, we mutated the tyrosine residue to alanine. This mutation reduced the glycerol permeability of AQP3 to 51 ± 15% of the wild-type permeability. In contrast, the urea permeability of the channel was increased from zero (or at least undetectably low) to 47 ± 20% of the wild-type glycerol permeability, giving a P_u_/P_g_ ratio of 0.93 ± 0.16. This provides experimental support for the idea of a glycerol-selecting hydrophobic corner in GLPs that preferentially conduct glycerol over urea. Together with the results for the H201A and H201G mutants discussed above, this provides the first experimental evidence that disruption of this hydrophobic corner can have a large effect on the glycerol:urea bias of the AQP pore.

Although the studies above support the role of the hydrophobic corner in selecting for glycerol, mutations in AQP4 aimed to mimic the ar/R-motif of GLPs (including the hydrophobic corner) failed to generate glycerol or urea permeable channels (Fig. 4). This, along with the differences between our AQP1 and AQP4 mutants, suggests that the exact residues of the ar/R region are not the only molecular determinant of solute permeability, but that the surrounding structural context is also important. This is in agreement with crystallographic analyses of AqpZ and GlpF, which suggested that positioning of the selectivity filter residues by the extracellular loops C and E may also contribute to AQP solute permeability/exclusion^10^.

We have, to our knowledge, made the first comparison of glycerol and urea permeability of human GLPs (AQPs 3, 9 and 10) in live mammalian cells, whilst controlling for relative surface expression. For AQP3, there are conflicting reports in the literature about whether it functions as a urea channel^19,20^ and it is not clear under what circumstances, if any, urea may permeate the channel. We have discussed this apparent discrepancy in detail in a recent review^14^. In our hands, human AQP3 was glycerol permeable, but was not measurably urea permeable when transiently expressed in HEK293 cells. To exclude artefacts associated with the use of GFP fusion proteins, we repeated measurements of AQP3 urea permeability in HEK293 cells expressing untagged AQP3 with the same result (data not shown). AQP10 had similar glycerol permeability to AQP3, whereas the permeability of AQP9 to glycerol was almost two-fold higher (Fig. 5). Interestingly, AQPs 9 and 10 had opposite biases towards glycerol and urea permeability with both having a P_u_/P_g_ ratio significantly different from an unbiased score of 1. It may be that these differences in the P_u_/P_g_ ratio represent a physiological mechanism by which the relative solute permeabilities of a membrane can be fine-tuned by altering the relative expression of different GLPs.

Plotting the urea permeability of the AQP4 mutants against the channel cross-sectional area revealed a linear relationship when the area was above ~14 Å2. This correlated well with the measured urea permeabilities of AQPs 3, 9 and 10, and the urea permeable AQP1 H180A/F56A mutant. Interestingly, the selectivity filter cross-sectional area of our modelled wild-type AQP3 structure is right on the threshold of permeability predicted by this model. We would therefore expect that if AQP3 is urea permeable, the permeability is very low. This may help to explain the conflicting data in the literature on AQP3 as a urea channel. In our hands it was not measurably permeable to urea, but this may be due to differences in timescale between our experiments (~1 min), and alternative approaches to measuring solute permeability, such as radiolabelled solute uptake. In contrast, no clear correlation could be found between glycerol permeability and cross-sectional area. This suggests that the selectivity filter geometrical dimensions are a key determinant for urea permeability but not for glycerol.

During preparation of this manuscript, an X-ray crystal structure of human AQP10 was published^21^. To estimate the reliability of our homology models, we therefore aligned our homology model based on GlpF to this structure (PDB code 6F7H). We find good agreement of the position of selectivity filter residues between our model and the crystal structure (selectivity filter residues heavy atom RMSD = 0.71 Å, see Fig. S1C) and the discrepancy between the selectivity filter radii calculated by HOLE and cross-sectional areas calculated by SYBL are <10% in both cases; we therefore proceeded with our homology model for *in silico* analysis. The quality of our prediction of the AQP10 channel geometry also lends weight to our homology models of AQP3 and AQP9.

Taken together, our data show that solute permeability of mammalian GLPs and solute exclusion by wAQPs depend on a complex interplay between the exact residues that form the ar/R region, the physical size and chemical properties of the filter created by these residues, and the structural context in which they are situated. For large protein families with high sequence, structural and functional homologies, the characteristics of model proteins may often be generalised to the whole family. Our study demonstrates that in the case of the AQP water channel protein family, this approach has led us to underestimate the complexity of the problem of water channel solute permeability. In order to develop a detailed understanding of water channel solute permeation, data on multiple members of the family should be compared and contrasted.

## Methods

### DNA constructs and mutagenesis

Human AQP cDNAs cloned into the C-terminal GFP expression vector pDEST47 were generated as previously described^22^. Untagged constructs were made by mutating the first two codons of the AQP-GFP linker peptide to two in-frame stop codons (TAGTGA). Success was confirmed by a ~25 kDa shift of the protein product in SDS-PAGE corresponding to loss of the GFP and lack of fluorescence after transient transfection into HEK293 cells. All site-directed mutagenesis was done using a modified QuikChange protocol, as previously described^23^.

### Calcein fluorescence quenching

10^4^ HEK293 cells were plated in black-walled, clear-bottomed 96 well plates (Corning) 48 h before experiments. 24 h after plating, cells were transfected using polyethyleneimine (branched, average Mw ~ 25,000, Sigma-Aldrich) and 150 ng DNA/well. All transfections were done in triplicate. On the day of the experiments, cells were washed once with growth medium (DMEM + 10% FBS, antibiotic-free) and incubated for 90 minutes at 37 **°**C with 5 μM calcein-AM (Molecular Probes, Life Technologies, diluted from a 5 mM DMSO stock) and 1 mM probenecid (an organic anion transporter inhibitor used to minimize calcein leakage from cells) in growth medium. After 90 minutes, cells were washed twice with HEPES-buffered growth medium with 1 mM probenecid to remove the DMSO used as a vehicle for calcein. Cells were then returned to the incubator for 10 minutes in HEPES-buffered growth medium (plus probenecid) to equilibrate with the new medium. The plate was moved to a pre-heated (37 **°**C) Biotek Synergy HT plate reader and calcein fluorescence was measured at 50 ms intervals for 10 s before and 50 s after injection of isotonic urea or glycerol solution, to a final permeant solute concentration of 150 mM. Osmolality of all solutions was measured using an Osomat 3000 freezing point depression osmometer (Gonotec).

### Cell surface biotinylation

Cell surface proteins from transiently transfected HEK293 cells were biotinylated and AQP surface expression was quantified using a neutravidin-based ELISA, as previously described^24^. For comparison between different AQPs, GFP-tagged constructs were used and GFP was detected in the ELISA using an anti-GFP antibody (Abcam ab6556, diluted 1:5,000).

### Permeability calculations

Normalised fluorescence data was converted to cell volume using a calibration curve derived from fluorescence intensity measurements and cell volume measurements (using a coulter counter) after equilibration with different concentrations of extracellular glycerol, adapted from Fenton *et al*.^12^. Exponential decay functions of the form C-Ae^−kt^ (with A the amplitude, k time constant and C = 1 + A), were fitted to the normalised cell volume data. Glycerol permeability was then calculated as P_gly_ = k(V_0_/A), with V_0_ the initial cell volume (estimated from the coulter counter calibration curve), and A the cell surface area (estimated as 9-fold higher than the surface area of a spherical cell of volume V_0_ to account for membrane folding, following Nemeth-Cahalan *et al*.^25^). By this method, we find that HEK293 cells expressing AQP3-GFP have a membrane glycerol permeability P_gly_ = 7.6 × 10^−6^ cm s^−1^, whereas GFP-transfected HEK293 cell have no measurable glycerol permeability (see Supplementary Fig. 2, panel C). For comparison between different aquaporins or different mutants, to account for variation in surface density we normalised P_gly_ to the surface density estimated from cell surface biotinylation ELISAs. All data is represented as surface density normalised P_gly_ (or P_urea_), as a proportion of the surface density normalised AQP3 P_gly_.

### Statistical analysis

For pairwise comparisons, unpaired *t* tests were used. For multiple comparisons, unpaired *t* tests were used following analysis of variance and *p* values subjected to Bonferroni’s correction. All *p* values referred to in the text and figures are the post-correction values, rounded up to 1 significant figure. *p* < 0.05 was considered statistically significant (*).

### *In silico* analysis

AQP4 mutant structures were generated based on the 1.8 Å AQP4 crystal structure^26^. Simulations were done using Gromacs software, version 4.5.5^27^. The GROMOS53A6^28,29^ force field was used and modified to include lipid parameters^30^. An AQP4 tetramer was generated according to the biological assembly entry in the AQP4 PDB file 3GD8^26^. Simulations were done as previously described^13^. Hydrogen bonds were identified using the Hbonds plugin of Visual Molecular Dynamics^31^ using 3 Å and 20**°** cut-offs. Hydrogen bond occupancy was calculated according to these cut-offs at 100 ps intervals along the trajectories and averaged over the four monomers.

A homology model of human AQP3 was generated using *E. coli* GlpF (PDB 1FX8) as template, using Swiss-Model^32^. *In silico* mutagenesis was done using Swiss PDB Viewer^33^ and the lowest energy amino acid sidechain conformer(s) were chosen in each case.

Channel radii of all structures were calculated using HOLE^34^ and channel cross-sectional areas using SYBL (Tripos Inc, St. Louis, MO, USA).

## Supplementary information

Supplementary figures.

## Data Availability

The datasets used and/or analysed during the current study are available from the corresponding author on reasonable request.
