# Supplementary material for: Water channel pore size determines exclusion properties but not solute selectivity
Source: Sci Rep. 2019 Dec 30;9:20369. doi: 10.1038/s41598-019-56814-z (PMC6937295; doi:10.1038/s41598-019-56814-z)
Supplement: Supplementary file 1 — Supplementary figures. [file 41598_2019_56814_MOESM1_ESM.pdf]

Water channel pore size determines exclusion properties but not solute selectivity

Philip Kitchen, Mootaz M. Salman, Simone U. Pickel, Jordan Jennings, Susanna Törnroth-Horsefield, Matthew T. Conner, Roslyn M. Bill and Alex C. Conner

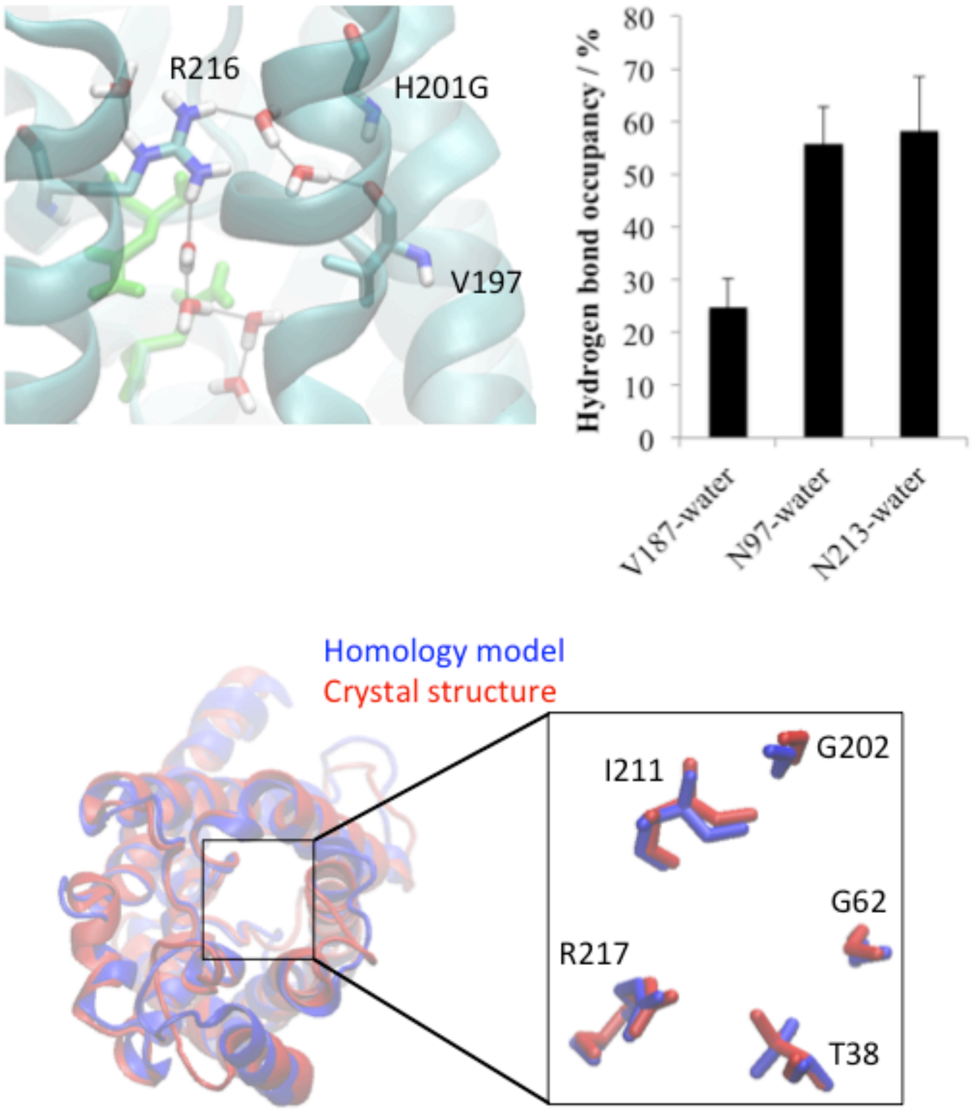

Supplemetary figure 1 - (A) Representative simulation snapshot of the AQP4 H201G mutant, showing the exposure of the V197 backbone to water molecules in the pore. Black lines represent hydrogen bonds. Asparagines of the NPA motif are coloured green. (B) Occupancy of the V197-water hydrogen bond, in comparison to the occupancy of the asparagine –water hydrogen bonds at the NPA motifs. (C) Alignment of x-ray structure of human AQP10 to our homology model of AQP10 based on GlpF.

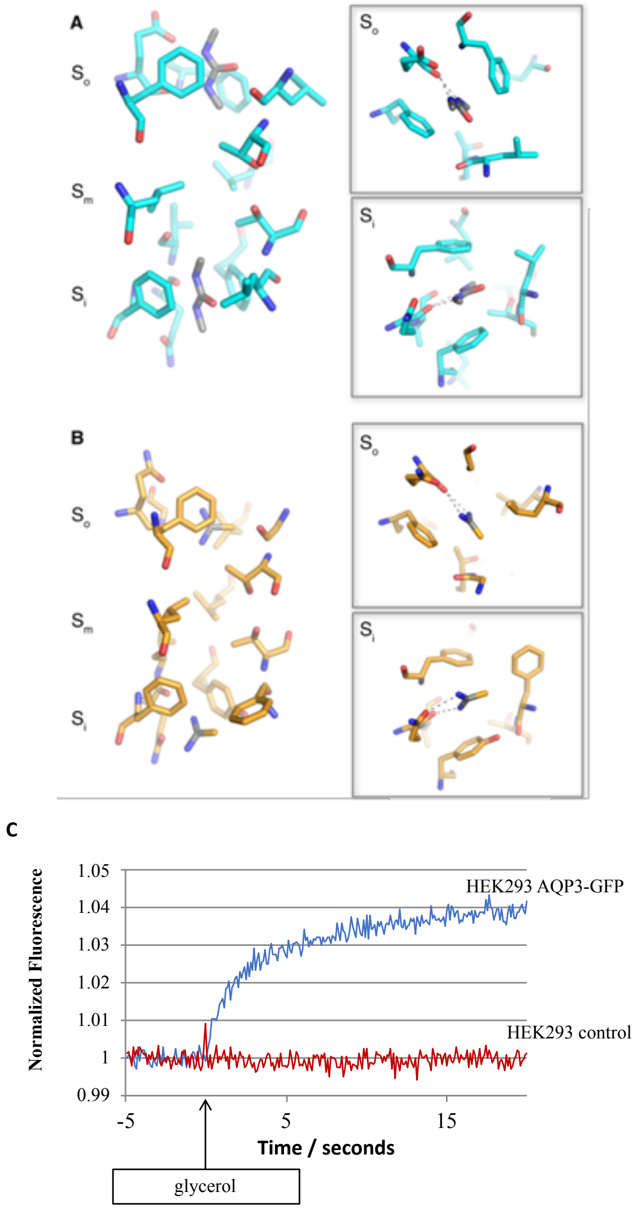

Supplemetary figure 2 – (A,B) Structural features of selectivity filter in urea transporters. Crystal structures of urea transporters from (A) Desulfovibrio vulgaris with dimethyl urea bound (PDB code 3K3G) and (B) Bos taurus with selenourea bound (PDB code 4EZD). In both structures, the selectivity filter is composed of an outer (S_o), middle (S_m) and inner (S_i) region and with dimethylurea/selenourea molecules bound within the the S_o and S_i regions only. (C) Representative calcein fluorescence timeseries from HEK293 cells transfected with AQP3-GFP or empty vector (control), and exposed to an iso-osmotic solution containing 150 mM glycerol, demonstrating low glycerol permeability of the HEK293 plasma membrane.
